# Emerging Paradigms in Genomics-Based Crop Improvement

**DOI:** 10.1155/2013/585467

**Published:** 2013-11-17

**Authors:** Abhishek Bohra

**Affiliations:** Indian Institute of Pulses Research (IIPR), Kanpur 208024, India

## Abstract

Next generation sequencing platforms and high-throughput genotyping assays have remarkably expedited the pace of development of genomic tools and resources for several crops. Complementing the technological developments, conceptual shifts have also been witnessed in designing experimental populations. Availability of second generation mapping populations encompassing multiple alleles, multiple traits, and extensive recombination events is radically changing the phenomenon of classical QTL mapping. Additionally, the rising molecular breeding approaches like marker assisted recurrent selection (MARS) that are able to harness several QTLs are of particular importance in obtaining a “designed” genotype carrying the most desirable combinations of favourable alleles. Furthermore, rapid generation of genome-wide marker data coupled with easy access to precise and accurate phenotypic screens enable large-scale exploitation of LD not only to discover novel QTLs via whole genome association scans but also to practise genomic estimated breeding value (GEBV)-based selection of genotypes. Given refinements being experienced in analytical methods and software tools, the multiparent populations will be the resource of choice to undertake genome wide association studies (GWAS), multiparent MARS, and genomic selection (GS). With this, it is envisioned that these high-throughput and high-power molecular breeding methods would greatly assist in exploiting the enormous potential underlying breeding by design approach to facilitate accelerated crop improvement.

## 1. Introduction

Plant breeding aims at tailoring the genetic architecture of a genotype in an artistic and scientific way, and its success is largely attributable to the extent of genetic variation present in the germplasm. The final outcome of all these breeding practices is an improved and publically accepted cultivar. Earlier methods based on direct or visual selection of phenotypes have contributed significantly in improving those commercially relevant traits which are governed by a limited number of major gene(s) or large effects quantitative trait loci (QTLs). Nevertheless, the traits controlled by a large number of smaller effects and epistatic QTLs and displaying significant genotype × environment (G × E) interactions could not be addressed appropriately through phenotypic selection (PS) based breeding methods [1, 2]. Within this context, accurate indirect selections based on genomic or molecular tools that have become prevalent over the last few decades have strengthened the traditional breeding to a great extent. In recent years, tremendous advancements have been made in the area of plant genomics leading to the dramatic increase in the number of genomic tools and technologies for almost every crop species [2]. For example, a wide array of marker systems has become available since the introduction of restriction fragment length polymorphism (RFLP) as the first genetic marker by Grodzicker and colleagues [3]. Importantly, this progress has been driven by next generation sequencing- (NGS-) based technologies and high-throughput (HTP) marker genotyping systems that have truly revolutionized the plant genomics [4]. In recent years, different kinds of rapid and cost-effective NGS-based sequencing technologies such as 454 FLX/Roche and Solexa/Illumina have been successfully employed for *de novo* whole genome shotgun (WGS) sequencing of reference genotype and whole genome resequencing (WGRS) of several cultivars, land races, and wild relatives [5, 6]. 

The remarkable progress has provided access to the plethora of genome-wide genetic markers especially single nucleotide polymorphism (SNP) markers which are particularly important from the aspects of throughput and automation [7, 8]. Additionally, recently available semi-/fully automated HTP genotyping systems have allowed accurate and rapid scoring of several hundreds to thousands of genetic markers. These include large-scale SNP genotyping systems like Illumina GoldenGate (GG)/infinium and moderate-scale assays such as MassARRAY, Taqman SNPlex, iPLEX, VeraCode, and KASPar assays [7]. Furthermore, due to recently introduced sequencing-cum-genotyping methods like restriction site associated-DNA (RAD) sequencing, genotyping-by-sequencing (GBS), and WGRS, a major shift has been revealed in the methods used for discovery and mapping of DNA markers [8, 9]. Notably, thousands of DNA markers could be discovered and mapped in a one-step process using these NGS-based methods [4, 9], thereby facilitating construction of high and ultrahigh density recombination maps not only for the major crop species with reference genome sequence but also for the crops where no reference genome is available [10].

In conjunction with the technological advancements, the concept of biparental linkage mapping is also changing to multiparent based mapping like multiparent advanced generation intercrosses (MAGIC) and nested association mapping (NAM) to enable reaping maximum benefits from the recently available HTP genotyping/sequencing and phenotyping platforms. The highly saturated recombination maps, thus developed for these populations would reveal the important genomic regions underlying economically important traits. Aside from traditional QTL mapping, these complex mapping resources create new possibilities for applying genome wide association studies (GWAS), and more importantly, joint linkage-LD analysis for a much comprehensible genetic investigation of complex traits [11, 12]. 

Remarkable changes have also been and are being witnessed in downstream deployments of the genetic markers/QTLs in crop improvement programmes. The conventional marker assisted selection (MAS) and the marker assisted backcrossing (MABC) programmes facilitate introgression of limited number of gene(s)/QTL(s). Though substantial genetic gains were achieved using MAS/MABC, issues related to minor QTLs could not be addressed compellingly through MAS/MABC approach [2]. Alternatively, marker assisted recurrent selection (MARS) scheme was proposed with the aim of accumulation of a number of QTLs into a single genotype. Taken together MABC and MARS schemes, however, target an individual marker or set of markers showing significant association with QTLs. Hence, still a considerable proportion of genetic variation remains unexplored [13]. To deal with this concern, a modification of MAS was proposed permitting selection of desirable genotypes on the basis of genome-wide marker information [14]. The method is referred as whole genome selection (WGS), genome wide selection (GWS), or genomic selection (GS) [15]. Keeping all these developments in view, this article provides a comprehensive review on these emerging molecular breeding approaches including their current status, impediments, and perspectives.

## 2. Biparental Genetic Populations: Trending towards Higher Mapping Resolution

Traditional mapping or family mapping focuses on generating experimental populations which are easy to establish and allow analysis of the genotyping and phenotyping data in a relatively simple manner. Mostly, these mapping populations are purposefully built, targeting a particular trait of interest. These populations are generated through crossing two genetically diverse parents and raising their F_1_ accompanied by selfing or backcrossing with the recurrent parent (RP) to achieve a segregating generation with a defined genetic constitution [11]. Following Collard et al. [16] mapping populations can be classified into two different categories, that is, ephemeral and immortal, and this classification is primarily derived from their genetic constitution, capacity to regenerate, and time required for establishment. Ephemeral or transient mapping populations harbour considerable proportion of heterozygous individuals within it, thereby making regeneration (with the same genetic constitution) practically impossible. The F_2_ and backcross (BC) populations represent such rapidly available genetic resources with almost half of the mapping individuals in the heterozygous state. However, the ease of generation and informative nature retain the major advantages associated with these mapping populations. 

By contrast, immortal populations are comprised of nearly homozygous individuals and thus represent “stable” resources which can be replicated over the years [16]. These include double haploids (DHs), recombinant inbred lines (RILs), near isogenic lines (NILs), advanced intermating lines (AILs), and so forth. Importantly, these populations are not affected by dominant/codominant nature of the marker system employed for genotyping. Concerning generation of these resources, DHs are developed with the help of embryo rescue techniques while RILs are developed through single seed descent (SSD) method. Generally, DHs represent a set of homozygous lines induced from F_1_ plants. However, based on simulation models a modified concept of “F_2_-derived DH” was proposed in maize to incorporate additional recombination events together with providing opportunities for practicing selections in segregating F_2_ generation [17]. 

Most investigations on genetic analysis of agriculturally important traits have been performed using biparental experimental populations. Accordingly, several softwares were developed for linkage and QTL analyses based on biparental populations (Table 1) [18–52]. Therefore, these populations have been the key resource for generating low- to high-density genetic maps and provided plenty of QTLs via gene tagging or QTL mapping [53]. Interestingly, biparental population still remains an ideal tool for detection of QTLs, and strictly, for the discovery of the rare alleles [54]. The major drawback with such populations, however, is the resolution of the identified QTL that is usually very poor [11, 55]. In other words, it assigns any QTL to larger intervals (broad chromosomal regions) [13, 51] thus making these QTLs unsuitable for future applications including map-based cloning or positional cloning. To redress this issue, enlarging the population size has been proposed as a viable option to enhance mapping resolution [16], but practically it is not possible to opt for several populations. 

Alternatively, increasing the chances for recombination events has been considered as more realistic and efficient means over increasing the size of mapping population [56]. With this view, an elaborated RIL approach was proposed as advanced intercross (AI) scheme permitting random matings among mapping individuals in F_3_, F_4_, and successive generations [11, 55, 56]. Relative to F_2_, RIL, and BC, high resolving power of AILs was evident from the fact that AIL is capable of mapping QTLs with the same precision that could otherwise be attainable through a three/four times larger F_2_ population [56, 57]. Therefore, AIL strategy empowers the traditional QTL analysis by incorporating extra rounds of intermating or genome reshuffling within reasonable population size. 

Besides, systematically built NILs, introgression lines (ILs), and chromosome segment substitution lines (CSSLs) enable fine mapping of QTLs; however, creation of such experimental populations is fairly cumbersome, and the entire procedure requires plenty of time. Traditionally, NILs are generated through repeated backcrossing with RP followed by selfing of the genotypes to genetically stabilize the improved versions. As an alternative, Tuinstra et al. [58] identified heterogeneous inbred family (HIF) as a relatively easy method to establish NILs. HIFs are generated by crossing two contrasting inbreds in a way similar to RIL development. Nevertheless, here segregation of the marker defined segments within family is monitored critically after F_5_ generation so that the NILs discriminating for the segment under consideration could be recovered. Using HIF approach, QTL-NILs differing for seed-weight were developed in Sorghum from F_5:8_ families [58]. Initially, RAPD markers were identified for the two seed weight related QTLs and subsequently within family segregation of these markers/QTLs was monitored using RAPDs. Similarly, HIFs have been developed in *Arabidopsis* from the cross “Bay-0 × Shahdara” aiming at detecting QTLs for root growth [59]. 

Since within a species, several mapping populations are developed at a time from multiple crosses targeting different traits. Therefore, a simple alternative to enhancing map resolution is merging the segregation data from multiple mapping populations to synthesize a much comprehensive genetic map known as “composite” or “consensus” maps. [23, 24]. The composite or consensus genetic maps harbour hundreds to thousands of loci offering greater genome coverage. For instance, the ultradense consensus genetic maps have been constructed for sunflower (*Helianthus annuus* L.) and cotton (*Gossypium* sp.), comprising 10,080 loci (and 1,310 cM) and 8,254 loci (with 4,070 cM), respectively [60, 61]. To make the consensus genetic map more informative, the QTLs identified from the component populations are also placed onto the consensus map, thereby increasing the chances for obtaining tightly linked and more informative DNA markers for QTL cloning or MAS and so forth.

More recently, with the gaining prevalence of NGS-based methods it has now become possible to perform high-resolution mapping with the moderate population size. For instance, WGRS facilitated development of ultra-high-density recombination maps for two rice RIL populations, namely “9311 × Nipponbare” and “Zhenshan 97 × Minghui 63” comprising 150 and 238 individuals, respectively [62, 63]. Similarly, highly saturated genetic maps were constructed for DH populations in wheat (Synthetic W9784 × Opata M85; 147 lines) and barley (Oregon Wolfe Barley (OWB); 82 lines) using GBS assays [10]. 

Furthermore, a novel mapping method has been invented for rice for rapid identification of markers tightly associated with the phenotype of interest. The strategy combines benefits of NGS and bulked segregant analysis (BSA) techniques. In this approach, a mutant phenotype is induced using EMS mutagenesis, and the induced mutant (in homozygous state) is then crossed to the wild type to generate a hybrid constitution (F_1_). The F_1_ is then selfed to give rise to F_2_ population showing a marked segregation for the mutant phenotype. Following this, DNA from mutant individuals in F_2_ populations are bulked, and the WGRS (up to 10 × coverage) for the bulked DNA is performed using appropriate NGS platforms. Finally, causative SNPs are detected through alignment of the generated sequence reads with the reference genome sequence [64]. Other similar approaches facilitating rapid gene discovery using NGS-based BSA include SHOREmap [65] and next generation mapping (NGM) [66].

## 3. GWAS: Emerging Approach to Scan Genome for QTL Discovery

Association analysis (AA) or LD analysis relies on exploring LD that is, the non-random association of alleles between different loci within genome [67, 68]. In addition to historical and evolutionary recombination events that have taken place during establishment of association panel, this non-random association of alleles is attributable to several other evolutionary forces such as mutation, domestication bottlenecks, genetic drift, and migration [68]. Unlike family-based QTL mapping that requires an appropriate segregating population and a genetic map, association or LD mapping harnesses genetic diversity existing among the naturally occurring diverse genotypes, thus circumventing the need for an experimental population [54, 55, 67–69]. In this manner, AA saves the time required to generate a mapping population together with enabling the use of the historical phenotypic data that has been recorded on diverse genotypes over the years [69]. Moreover, linkage mapping provides QTLs that are mostly population specific, whereas AA tests multiple alleles for their association with the trait, therefore making the later more realistic for QTL discovery. A list of softwares used for LD mapping or AA has been provided in Table 1.

The extent of LD decay across the genome primarily decides the number of DNA marker required to extract meaningful inferences. In general, cross-pollinating species exhibit lower levels of LD or higher levels of LD decay than the self-pollinating species [54]; therefore, comparatively higher number of DNA markers would be required in case of cross-pollinating species to unravel the molecular mechanism of any complex trait. However, variations in the levels of LD decay have also been reported within species and from locus to locus within a particular genome [67]. For example, in case of maize (a cross-pollinated species), LD decays rapidly over 1 kb in landraces and 2 kb in diverse inbred lines while, in case of commercial elite inbred lines, it extends up to 100 kb. In contrast, LD extends up to 250 kb in *Arabidopsis* (a self-pollinating species) [70]. 

AA can further be classified into two categories: a candidate gene approach that targets genotyping of specific genomic region. Contrary to it, another approach known as GWAS requires genome-wide markers and scans the entire genome for detection of QTL signals [54, 55]. During the initial phase, when genotyping and sequencing were prohibitively-costlier, candidate gene approach, requiring less number of markers was considered more suitable. Nevertheless, with the rapidly declining genotyping/sequencing cost and availability of high-density genetic maps/haplotype maps (HapMaps), GWAS has rapidly emerged as an appropriate tool for the identification of genetic variants associated with important traits. For example, recently a total of 950 worldwide rice cultivars were chosen to apply GWAS to discover the important loci underlying flowering time (heading date) and grain-related traits, and, consequently, 32 novel loci were detected [71]. Similarly, whole genome scans were also employed in several other crops like maize [72], wheat [73], *Arabidopsis* [74], barley [75], sorghum [76], and so forth. GWAS is a powerful means for delivering precisely mapped QTLs and offers an obvious way to cross-validate the QTL results obtained from family-based QTL mapping. Another attractive feature of LD analysis is that it enables the genetic analysis of multiple alleles and multiple traits at a time which is otherwise restricted to two alleles and limited traits in case of biparental trait mapping [68, 69]. 

Contrary to linkage mapping, AA is relatively inefficient in capturing the rare variants [50, 54, 55, 68]. Furthermore, the major operational bottleneck in AA is the difficulties arising due to the population stratification or, precisely, the population structure that often leads to the establishment of spurious linkages even between the unlinked loci, that is, false positives [11, 55, 69, 77]. However, rate of generation of false positives depends on the phenotypes as well. For example, flowering-time related phenotypes exhibit more spurious associations because of the distinctive geographical distribution of these phenotypes [59]. The detection of false positives may inflate as high as 40% in case of GWAS [55]. Though, several methods have been developed to control false positives such as transmission disequilibrium test (TDT), principal component analysis (PCA), genomic control (GC), structured association (SA), and unified mixed model approach (Q + K) [68, 70], these methods are vulnerable to lose some of the true/potential QTLs (termed as false negatives) [77]. Concerning the extent of false negatives, reports have been published indicating that sometimes the frequency of false negatives may be alarmingly high, that is, up to 25% [55] or even 40% [59]. Within this context, the multiparent derived experimental populations like NAM and MAGIC with greater allelic richness and no population structure are considered to be promising tools for GWA studies [68, 77].

## 4. Next-Generation Genetic Populations: High-Power Mapping Resources for Community Research

Precise mapping of QTLs is directly related to the frequency of recombination, which in turn depends on frequency of intermating between the founder genotypes as well as among the mapping individuals [54–57]. With this consideration, the idea of developing heterogeneous stock (HS) was conceived in mice [78]. In HS approach, multiple parents are allowed to intermate in a pairwise fashion for several generations. Therefore, HS model was successful in narrowing down a broad QTL region to the level of few genes. However, the major problem experienced with HS population is a requirement for repeated genotyping owing to its highly heterozygous and heterogeneous nature [50]. Taken into consideration the above issue of genomic mosaics, a novel software package “HAPPY” was developed to perform multipoint QTL mapping using HS [78].

Further, to make provisions for replicated measurements, a modified version of HS scheme was developed as collaborative cross (CC) mating system under the umbrella of the complex trait consortium (CTC) [79]. Collaborative cross is an integrated mapping approach which was specifically intended to deliver a global resource for dissecting the genetic architecture of complex traits. CC consisted of RI strains generated through crossing of genetically diverse founders capturing considerable amount of known genetic variation. Due to RI constituents, CC is likely to be a reproducible genetic resource specifically suited for investigating molecular networks, epistatic interactions, and trait correlations that collectively define the complex biological system [79]. The intrinsic resolution power of CC-RI strains has already been documented [80, 81]. Similarly, with the objective of generating high-resolution mapping resources, the concept of multiple founders was also adopted in plants in the form of MAGIC and NAM [11, 82]. A brief comparison among different types of biparental and multiparental populations has been demonstrated in Table 2. 

MAGIC populations are being developed in various crop species, and exciting results have already been published from MAGIC populations in *Arabidopsis*, wheat, and rice. The first comprehensive MAGIC panel in plants was reported for *Arabidopsis* in which a total of 19 founder accessions were used to develop over thousand MAGIC lines (MLs) [83]. Of these, a set of 527 MLs was chosen to perform a high-power and high-resolution QTL mapping for developmental traits such as days to bolt, days to flower, and growth rate [50]. Another *Arabidopsis* MAGIC population, popularly known as *Arabidopsis* multiparent RIL (AMPRIL), was derived from eight accessions which were crossed to produce a total of four two-way hybrids, and the resultant hybrids were then mated in a diallel fashion [84].

In a similar manner, a MAGIC population consisting of 1,579 progenies was developed for wheat at CSIRO, Australia. Four Australian wheat genotypes (Yitpi, Baxter, Chara, and Westonia) acted as founder parents to build a 4-way MAGIC panel. Furthermore, this MAGIC population was used to yield a high-density genetic map for wheat representing the first MAGIC map in any plant species. The MAGIC map spanned a total of 3,894 cM with 1,162 marker loci [51]. 

MAGIC approach has been implemented in rice at a much larger scale. A total of four multiparent populations namely *indica*-MAGIC, MAGIC-plus, *japonica*-MAGIC, and Global-MAGIC were developed to offer additional insights on tolerance to submergence and bacterial blight [85]. The *indica*-MAGIC comprises of eight *indica* parents, while *japonica*-MAGIC contains eight founder genotypes from *japonica* rice. Furthermore, MAGIC-plus is an extended *indica*-MAGIC encompassing two extra rounds of intermating whereas Global-MAGIC represents an excellent attempt to capture the diversity in both *indica* as well as in *japonica* rice. All the sixteen genotypes used for creation of *indica*- and *japonica-*MAGIC were chosen as parents for Global-MAGIC population [85]. 

Like MAGIC, NAM represents another powerful public resource, comprising various RI populations sharing genome of one of the parental genotypes. For example, the NAM design in maize was developed by crossing 25 diverse genotypes to a common parent B 73 (a popular inbred line of maize) to create a total of 25 different RI families connected with each other through shared ancestry [86]. Technically, this purposely designed mating scheme in NAM led to the development of ~5000 RI lines capturing large proportions of the genetic diversity existing in maize [82]. 

Further, to make NAM design cost effective and more striking, a different strategy was applied for genotyping of RI individuals. It is important to note that, in maize NAM, emphasis was given towards the discovery of those alleles which were specific to “B 73,” and, accordingly, these markers were referred as common parent specific (CPS) markers [86]. Subsequently, both genotyping strategies, that is, typing all SNPs or only CPS-SNPs, were compared, and an inference was drawn that both methods yielded more or less similar experimental results.

Notably, several GWAS and joint linkage-LD analysis were performed using NAM in maize [87]. For example, an investigation on genetic architecture of starch, protein, and oil kernel composition in maize revealed several smaller effects QTLs, of which half of the QTLs were reported in previous studies [88]. In addition, NAM provided a detailed genetic analysis of *DGAT1-2* genomic region covering approximately 25 Mb in the genome [88]. Similarly, another important trait in maize, that is, leaf architecture, was subjected to GWAS using NAM permitting identification of the underlying genes/QTLs [87]. Moreover, by extracting SNPs from Maize HapMap, NAM-GWAS was undertaken in maize to find out the genetic determinants conditioning resistance against southern leaf blight disease [89].

Given immense success of NAM design in maize, similar mating scheme was opted in *Arabidopsis* for genetic analysis of flowering time in a set of thirteen RIL populations [59]. Of these thirteen RILs, twelve shared a common parent, that is, “Columbia” (Col-0), a widely used wild accession. The remaining RIL “Bay-0 × Sha” was also used for development of HIFs and NILs to further validate the identified QTLs. The entire experiment included a total of 4,366 individual RI lines. The detailed information on the RIL populations is made publically available at the Versailles *Arabidopsis* Stock Center (http://publiclines.versailles.inra.fr/). Interestingly, these populations provided over sixty QTLs for flowering time with the PV ranging from 30 to 60%.

Another *Arabidopsis* NAM population constituted of three biparental RIL families, namely “Ler × An-1”, “Ler × Kas-2” and “Ler × Kond” [12]. A new algorithm of joint inclusive composite interval mapping (JICIM) was proposed in this study. Interestingly, JICIM outperformed the traditional QTL mapping since all the QTLs which were present in individual RIL populations were detected with stronger evidences (at very high LOD values). Notably, the ability of NAM population for detecting rare QTLs was also demonstrated experimentally through conducting a comparative study between JICIM and individual family-based QTL mapping [12]. 

Recently, a modified version of NAM, backcross derived NAM (BCNAM), has been initiated in sorghum for improving the quality and the yield in West Africa by IER, CIRAD, and ICRISAT (http://www.generationcp.org/sorghum-bcnam-project-2). The BCNAM design involves three popular cultivars as RPs which would be crossed to ten specific donor parents (SDP) and ten common donor parents (CDP) to generate a set of backcross populations.

## 5. Genomics-Assisted Introgression Breeding Using Exotic Germplasm

Modern-day varieties in any crop species are products of several human-mediated processes, or more appropriately, the domestication bottlenecks [90, 91]. Surprisingly, only a small fraction of the entire gene pool is exploited during the development of cultivars which may have higher productivity and adaptability, but at the cost of valuable genetic diversity [91]. The situation is more unfavourable for self pollinated crops because it has been found that during the development of modern-day cultivars considerably large proportion of natural variation (nearly 95%) has remained untouched [92]. 

Wild relatives or landraces in various crop species represent large, natural, and underutilized pool of vast genetic diversity which could better be explored for the identification and introgression of favourable exotic alleles into the elite breeding lines. Therefore, revealing the key genomic regions associated with the domestication process. Taken into consideration, a mapping strategy was designed by Tanksley and Nelson [93], focussing on extraction of the genomic information from wild and unadapted genotypes such as wild ancestors and landraces. The scheme was referred as advanced backcross QTL (AB-QTL). In addition to discovery of superior exotic alleles, this approach helps in expanding the genetic base of the cultivated gene pool. 

**Figure 1:**
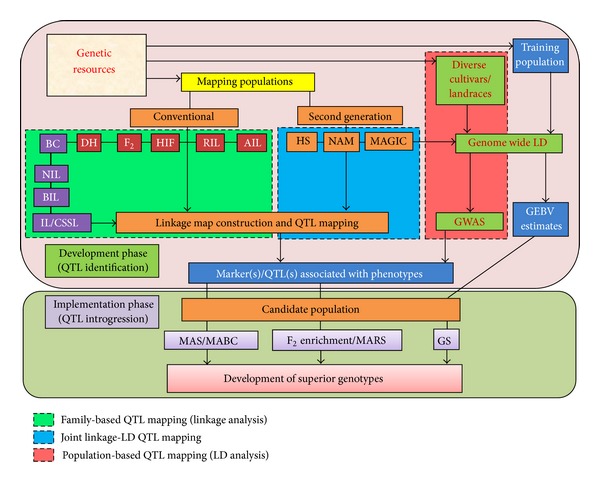
Schematic representation of genomics assisted crop improvement. ∗DH: Double haploid; BC: Backcross population; RIL: Recombinant inbred line; AIL: Advanced intercross line; HIF: Heterogeneous inbred family; NIL: Near isogenic line; BIL: Backcross inbred line; IL: Introgression line; CSSL: Chromosome segment substitution line; HS: Heterogeneous stocks; NAM: Nested association mapping; MAGIC: Multiparent advanced generation intercross; MAS: Marker assisted selection; MABC: Marker assisted backcrossing; MARS: Marker assisted recurrent selection; GWAS: Genome-wide association study; GS: Genomic selection.

AB-QTL method has several advantages over traditional linkage mapping. Generally, linkage mapping represents the developmental phase in which marker-trait associations are discovered. Practical implications of these marker-trait relationships, however, are realized during the next phase, that is, trait introgression (Figure 1, Table 3). Conversely, AB-QTL is an integrated mapping strategy in which both procedures, namely “mapping” and “transfer,” are executed within the same population, which is usually a backcross population derived from a wide cross [92]. 

The entire procedure inherently avoids the possibilities for building unpredictable interactions with the new genetic background that otherwise poses hindrance in anticipated expression of the introgressed trait. Here, QTLs are identified in the advanced generations rendering QTLs with only additive effects and thus eliminating chances for establishment of epistatic interactions [94]. For betterment, selection is practised against undomesticated traits like shattering thus allowing progression of only favourable exotic alleles to the advanced generations. Additionally, a valuable byproduct of this method is rapid and systematic generation of QTL-NILs [92]. 

Further, availability of high-density marker information has guided precise tracking of exotic chromosomal segments leading to the availability of exotic libraries [94]. Exotic genetic libraries are comprised of a series of ILs/CSSLs collectively covering entire genome of the donor parent [95]. Well-characterized ILs/CSSLs have been reported in several crops like tomato [95], rice [96], barley [97], and so forth. In tomato, an exotic library composed of 76 lines was constructed using drought-tolerant wild species *Solanum pennellii* as donor and elite inbred variety M 82 as RP. Similarly in rice, 128 CSSLs were developed from the cross between *indica* (9311) and *japonica* (Nipponbare) genotypes [96]. Moreover, to assist *in silico* development of CSSL lines, softwares like CSSL Finder have also been introduced (https://www.integratedbreeding.net/supplementary-toolbox/genetic-mapping-and-qtl/cssl-finder). 

## 6. F_**2**_ Enrichment and MARS: Potential Methods to Incorporate Multiple QTLs

Introgression of the QTLs into another genetic background is the most important step in molecular crop improvement because of its direct relevance to the development of improved cultivars. An inclusive genomics-based approach for trait introgression has been illustrated in Figure 1. Among several methods being used for trait introgression, backcrossing is a well-established method routinely used for introgression or defect elimination, but its progress as well as accuracy is hampered by (i) slow decrease rate of undesirable donor genome or linkage drag and (ii) time taken for the maximum recovery of the RP genome. Theoretically, based on the formula BC_*n*_ = (2^*n*+1^ − 1)/2^*n*+1^, recovery of RP genome after any *n*th backcross generation can be predicted; however, some plants may possess more or less than the expected percentage of RP genome [16]. Markers based foreground selection especially recombinant selection is performed for precise transfer of donor genome resulting in minimization of linkage drag. In parallel, background selection or selection against the donor genome is practiced to maximize the RP genome recovery in each backcross with the help of the markers that are unlinked to the target locus [98]. Marker assisted foreground and background selections offer much faster elimination of the undesirable alleles that are associated with the genomic fragment of interest [4]. Like traditional backcrossing, the final outcome of MABC is an improved version of existing popular cultivar. Given the ability to transfer major QTL(s)/gene(s), MABC is particularly useful for stacking of genes conferring strong and durable resistance [4, 53], but pyramiding is usually inefficient for quantitative traits (QTs) controlled by several QTLs with variable phenotypic effects [2]. 

In addition to linkage drag, another potential obstacle in trait introgression is pleiotropy often causing correlated response (indirect selection for nontargeted trait). Molecular dissection of this complex phenomenon has revealed that pleiotropy may result from intragenic linkages between quantitative trait polymorphisms (QTPs) [99]. Like gene *dwarf 8* in maize which controls both flowering time and plant height, but both these activity are regulated by two different *SHT2* and *DELLA* domains, respectively *via* two separate QTPs [99]. As recombination within gene is not desirable thus emphasis should be given for detection of haplotypes combining favourable QTP alleles for both traits. In this way understanding of pleiotropy at gene level would help in avoiding unnecessary efforts given for recovery of recombination events required to break undesirable linkages. 

In terms of complexity of traits, MARS is more relevant than MABC because the former is able to harness even those QTLs experiencing minor effects on the phenotype. Concept of MARS has been borrowed from conventional recurrent selection, a scheme proposed by Hull [100]. Phenotypic recurrent selection has been one of the potential methods for population improvement involving repetitive cycles of selfing, intercrossing, and selection [1]. Recurrent selection scheme has contributed significantly in improving response to selection in both self- and cross-pollinated crops such as maize and soybean [101]. Nevertheless, extremely long cycles and repeated phenotyping are the major barriers hampering its extensive use in breeding programmes. As a refinement, integration of DNA marker technology with the traditional recurrent selection was advocated, and, consequently, modern theory of MARS came into existence in which individuals in F_2_ or any other derived generation are initially selected through analyzing the phenotype and marker data [102]. Whereas in the later generations, desirable genotypes are selected using marker scores, that is, exclusively marker data based selections. Marker scores for any individual are calculated by a formula given by Bernardo [53]. Finally, the selected individuals are allowed to recombine for the next two to three generations. 

Therefore, MARS favours speedy development of superior “mosaic” genotypes by extracting superior alleles from both parents [103] through a procedure similar to the F_2_ enrichment. In F_2_ enrichment, selection is practised against negative homozygous alleles in F_2_ population, thereby increasing frequency of superior alleles in the form of homozygotes/carrier heterozygotes in the advanced generations. Concerning changes in allelic frequency after F_2_ enrichment, it has been found that, for ten QTLs, F_2_ enrichment changes the frequency of favourable allele from 0.50 to 0.67 [53]. F_2_ enrichment involves only one generation of marker based selection. Hence, frequency of superior allele attainable through F_2_ enrichment may not be sufficient to meet expectations when the PV is accounted to numerous QTLs [53].

Given the ability to capture multiple QTLs, MARS has several advantages over PS, MABC, and F_2_ enrichment. Empirical and simulation results have indicated that response to MARS is found to be superior to MABC and phenotypic recurrent selections [103]. MARS led to 3% to 20% enhancement in genetic gains than PS [104], whereas, in terms of change of frequency, MARS increased the frequency of the favourable marker allele from 0.50 to ≥0.80 in a sweet corn F_2_ population [53]. Unlike MABC, MARS does not essentially need a preestablished QTL-phenotype relationship because QTL mapping can be performed within MARS scheme itself to recover the existing QTLs, or more appropriately, an *ad hoc* significance test can be conducted [104]. Nevertheless, it is reported that the response to MARS increases with the prior knowledge of the QTLs [104]. On the other hand, unknown QTLs could be discovered and incorporated in MARS by identification of the markers associated with the trait and the effect of these markers on the trait. 

MARS dramatically enhances the probability of recovering the superior genotypes possessing combinations of the favourable alleles [2]. For instance, Eathington et al. [105] have reported that, for 20 different QTL regions, a change in frequency of favourable allele from 0.50 to 0.96 significantly enhances the probability of recovering an ideal genotype from one in a trillion to one in five. In another notable example, MARS enhanced the gains by twofold when employed in maize breeding populations as compared to PS [106]. MARS has been emphasized in private sectors like Monsanto and Syngenta for the improvement of maize, soybean, and sunflower [105]. However, encouraged by the above successful instances, MARS is being extended to other crops including rice, sorghum, chickpea, common bean, and cowpea with the help of CGIAR and various NARS centres (http://www.generationcp.org).

## 7. Genomic Selection (GS): A Genome-Wide High-Throughput Approach to Predict Performances

Traditionally, breeding value (BV) has always been an important indicator routinely used for assessment of practical worth of any given genotype [14]. BV of any individual is defined as a value obtained from the average performances of its progenies. Best linear unbiased predictions (BLUPs) based on phenotypic data are routinely used to calculate the estimated breeding values (EBVs), and selection is practiced on the basis of these EBVs [15, 107]. With a similar idea of using genome-wide marker data for prediction of performance, Meuwissen et al. [15] proposed GS scheme in animals that tests thousands of DNA markers to derive estimates of BVs for each genotype, known as genomic estimated breeding values (GEBVs). As BVs are dependent on the magnitude of additive effects, GEBV-based GS models exploit additive effects operating within a population [52]. 

Conventional MAS/MABC approaches normally utilize the major effect QTLs, and consequently substantial degree of variation accounted to small-effects QTLs remains unaddressed [14]. Secondly, the QTL mapping methods are prone to losing genomic regions playing important roles in manifestation of complex traits [13]. By contrast, GS targets hundreds/thousands of DNA markers at a time that are in strong LD with the genomic regions of interest. The idea underlying the GS scheme is that, in comparison to a single marker, haplotypes offer greater possibilities to be in LD with a particular QTL [15]. In this way, GS operates at whole genome level without searching for significant individual marker-trait relationships. A precise comparison of various molecular breeding schemes has been made in Table 3.

GS scheme uses “training population” as a base constituent that actually serves as model since individuals from training population are subjected to genome-wide genotyping and extensive phenotypic evaluation [15, 107]. Since it provides estimates of the marker effects through utilizing genome wide marker information, therefore, critical attention has to be paid while designing training population. On the other hand the “candidate or breeding population” acts as a platform for selecting individuals on the basis of the sum of BVs across all the markers [14, 15]. In other words, no additional phenotyping is required for candidate population. GS lessens time duration and cost by eliminating the need for repeated phenotyping, and QTL mapping. However, for improving the practical usability of GS, inclusion of another population (described as validation population) has also been advocated [14, 52].

Concerning the composition of the training population, different kinds of populations have been tested in various simulation and empirical studies [108]. These populations included biparental mapping populations like F_2_, RILs, DHs, sets of diverse inbred lines and full sib families, and so forth. Furthermore, populations derived from multiparental mapping systems like NAM have also been considered as potential test populations for deriving GEBV predictions. However, the accuracy with which GEBVs could be predicted depends on several other factors like population size, number of markers, and the relation between training and breeding populations [52]. 

Furthermore, the choice of appropriate statistical model for prediction of GEBV would likely be a crucial factor in determining the success of GS. Various algorithms have been optimized for GS prediction like ridge regression, Bayesian based [BayesA, BayesB, weighted Bayesian shrinkage regression (wBSR), Bayesian least absolute shrinkage and selection operator (LASSO)], random forests (RF), and support vector machines (SVMs), and effectiveness of these methods have already been compared in several studies [109–111]. Interestingly, Iwata and Jannink [108] performed a simulation study with more than 800 barley lines using approximately 1,000 SNPs and concluded that the average of several models provided more accurate estimates than the individual model particularly in context of low to moderately heritable traits. Additionally, the extent of LD decay between the markers and the target genomic region also affects the accuracy of GS [110]. 

Unlike regular breeding programmes, the major objective of phenotyping in GS is to predict GEBVs rather than selection of genotypes [14, 107]. Promising genotypes, however, are selected later on the basis of GEBV estimates. Several simulation and empirical studies have been published on GS relating to accurate prediction of GEBV estimates and the relative advantages to other marker based selection schemes [112–114]. In maize DH line, it was observed that response to GS was 18 to 43% higher than MARS across different levels of population sizes, numbers of QTLs, and levels of heritability. Specifically, higher response was more evident in the case where the trait was governed by QTLs with low heritability [115]. Similarly, Wong and Bernardo [116] reported that GS could result in the release of improved germplasm in oil palm within six years in contrast to 19 years generally taken through PS. More recently, a report on GS has been published on fruit-quality traits in apple. Given the low heritability of traits, gains in GS were found to be almost 100% higher than the conventional BLUP based selection models [110].

By its nature, GS focuses on genetic improvement of QTs rather than understanding their genetic basis [52, 115]. In addition to identify a large number of small-effects QTLs scattered throughout the genome, this approach can also be applied for the selection of potential parental lines thus escaping rigorous phenotypic assessment in the target environments [107]. Being in extrapolatory phase in plant science, practical examples of GS are not adequate, but the preliminary analyses look promising and emphasize that the success of GS in plant science would largely depend on the extent of accuracy in GEBV predictions. 

## 8. Opening Rich Opportunities for Practising Breeding by Design

Ideotypes or ideal plant types are known to plant breeder, since 1968 when Donald [117] defined an ideotype as a hypothetical biological model designed to perform in a predictable manner under defined environmental conditions. Based on morphology and physiology, ideotypes have been suggested in many crops including barley, wheat, rice, and so forth. Although this concept could not provide likely gains practically, it has always played a major role in framing various crop breeding strategies [101]. In the post-genomics era, the concept of ideotype has been taken to the next level where designing of ideal genotypes could be performed *in silico* and Peleman and van der Voort [118] described it as breeding by design. 

The concept of breeding by design includes (i) locating genes/QTLs associated with important traits (ii) exploring the allelic variation at these loci and the estimation of phenotypic effects of these allelic variants (iii) choosing desirable recombinants by targeting marker/haplotype-defined genomic fragments [4, 118]. Recently, several softwares and tools like ISMAB(information system for marker assisted backcrossing) have also been developed to support *in silico* designing of a superior genotype through combining desirable loci (https://www.integratedbreeding.net/ib-tools/breeding-decision). 

Availability of highly saturated genetic maps and populations like ILs has facilitated fine mapping of various QTLs [97]. Further, growing emphasis on multi-parent mating systems offer rich opportunities for precise mapping of the QTLs [50, 51, 85]. These lines harbouring diverse alleles at the loci of interest can be phenotyped accurately to give an idea about the phenotypic values of these alleles. In case of exotic genetic libraries, epistatic interactions among various QTLs could be estimated by combinations of these introgression lines with different QTLs and different genetic backgrounds [94]. 

Once genetic loci influencing the expression of the trait have been mapped precisely, allelic variants at all these loci can be mined along with their relative contribution to complex traits, and highly resolved marker haplotypes could be recovered for several agriculturally important traits [118]. With the help of accurate phenotyping measurements, one can have better idea about the phenotypic effects of all the allelic variants and subsequently, predictive improvement [82] could be performed in a way that would ensure the highest probability for recovering the genotypes with desirable haplotypes or allelic variants.

Moreover, precise phenotyping or phenomics is one of the major bottlenecks in capitalizing the full potential of breeding by design concept [118]. Therefore, tremendous attention is being paid towards establishment of automation-driven, cost-effective, and robust phenotyping systems [119, 120]. For instance, recently available HTP platforms like LemnaTec scanalyzer3D [121] and RootReader2D/3D [122]. Further, some of the phenomics platforms/software tools that are being used in precise phenotyping are listed in Table 4 [119–139]. Easy access to such phenotyping facilities would definitely encourage researchers for making breeding by design a routine practise in genomics based crop improvement schemes. 

## 9. Conclusion

Rapidly decreasing genotyping and sequencing costs are dramatically changing the scenario of genomics-assisted breeding. For instance, a shift has been seen from biparental to multiparental populations, and, with the help of various NGS-based sequencing platforms, a detailed genetic analysis of these complex mapping resources would likely to be feasible. Moreover, extensive recombination and multiallelic nature of these lines make them an excellent platform for practising multiparent MARS and GS. More importantly, the development of such public resources like MAGIC and NAM would strengthen the community-based research approach [11]. 

Additionally, by virtue of eliminating need for any prior QTL information, MARS and GS schemes would save time, money, and energy that is required for finding significant gene-trait relationships. Still, realization of immense potential of all these approaches would greatly rely on throughput, precision, and cost effectiveness of phenotyping techniques. Though, precise phenotyping has always been a potent limiting factor in genetic analysis of QTs, efforts are underway to meet the growing demands for accurate and HTP screening against various biotic/abiotic stresses. It is envisaged that parallel developments in the next-generation phenotyping systems would help in making GS a practical reality in case of plant species as well. Therefore, rising molecular breeding methods like MARS or GS would enable harnessing unexplored genetic variation to a greater extent, thereby facilitating speedy development of superior cultivars.

## Figures and Tables

**Table 1:** List of softwares used for various analyses in molecular breeding.

Name of software	URL	Reference
Linkage map construction		
MAPMAKER/EXP	http://www2.hawaii.edu/~durrell/Software/B14F5F09-6238-43DA-B95F-958D4FB709AD.html	[18]
JoinMap	http://www.kyazma.nl/index.php/mc.JoinMap	[19]
RECORD	http://www.wageningenur.nl/en/show/RECORD.htm	[20]
AntMap	http://lbm.ab.a.u-tokyo.ac.jp/~iwata/antmap/	[21]
MST_MAP _	http://alumni.cs.ucr.edu/~yonghui/mstmap.html	[22]
MergeMap	http://138.23.178.42/mgmap/	[23]
MultiPoint	http://www.multiqtl.com/	[24]

Family based QTL discovery		
MAPL	http://lbm.ab.a.u-tokyo.ac.jp/~ukai/	[25]
MapQTL	http://www.kyazma.nl/index.php/mc.MapQTL	[26]
PLABQTL	http://wheat.pw.usda.gov/jag/papers96/paper196/utz.html	[27]
QGene	http://www.qgene.org/qgene/index.php	[28]
BQTL	http://famprevmed.ucsd.edu/faculty/cberry/bqtl/	[29]
Map Manager QTX (QTX)	http://www.mapmanager.org/	[30]
Windows QTL Cartographer	http://statgen.ncsu.edu/qtlcart/WQTLCart.htm	[31]
MCQTL	http://carlit.toulouse.inra.fr/MCQTL/	[32]
GMM	http://www.kazusa.or.jp/GMM/	[33]
ICIM	http://wiki.cimmyt.org/confluence/display/MBP/Application+2.2.5+Tool+7.10+QTL+IciMapping	[34]
QTLNetwork	http://ibi.zju.edu.cn/software/qtlnetwork/	[35]
R/qtl	http://www.rqtl.org/	[36]
MultiQTL	http://www.multiqtl.com/	—

LD analysis (Population structure/Marker-trait association)		
STRUCTURE	http://pritch.bsd.uchicago.edu/software/structure2_2.html	[37]
EIGENSTRAT	http://www.mybiosoftware.com/population-genetics/1309	[38]
GeneRecon	http://www.daimi.au.dk/~mailund/GeneRecon/	[39]
GENOMIZER	http://www.ikmb.uni-kiel.de/genomizer/	[40]
BMapBuilder	http://bios.ugr.es/BMapBuilder/	[41]
CaTS	http://www.sph.umich.edu/csg/abecasis/CaTS/	[42]
MIDAS	http://www.genes.org.uk/software/midas	[43]
TASSEL	http://sourceforge.net/projects/tassel/	[44]
InStruct	http://cbsuapps.tc.cornell.edu/InStruct.aspx	[45]
PLINK	http://pngu.mgh.harvard.edu/~purcell/plink/	[46]
GenAMap	http://cogito-b.ml.cmu.edu/genamap/	[47]
GWAPP	http://gwas.gmi.oeaw.ac.at/#!home	[48]
ALDER	http://groups.csail.mit.edu/cb/alder/	[49]

MAGIC analysis		
R-version of HAPPY	http://www.well.ox.ac.uk/happy/happyR.shtml	[50]
mpMap	http://www.mybiosoftware.com/population-genetics/6437	[51]

Genomic selection		
R-Package for GS	http://www.r-project.org	[52]

**Table 2:** Comparison among various genetic populations.

	F_2_	DH	BC	RIL	NIL	AIL	MAGIC	NAM
Parents involved	Two	Two	Two	Two	Two	Two	Multiple	Multiple
Resource Type	Transient	Immortal	Transient	Immortal	Immortal	Immortal	Immortal	Immortal
Mapping individuals	Heterozygous and homozygous	Homozygous only	Heterozygous and homozygous	Homozygous only	Homozygous only	Homozygous only	Homozygous only	Homozygous only
Generations required	Two	Two	Two	Six to eight	Six to eight	Usually ten	More than eight	More than six
Multiple alleles/multiple traits	No	No	No	No	No	No	Allowed	Allowed
Recombinant events	Limited	Limited	Limited	Limited	Limited	High	High	High
Suitable for	Family-based linkage (FBL) mapping	FBL mapping	FBL mapping	FBL mapping	FBL mapping	FBL mapping	Dual linkage-LD	Dual linkage-LD
Mapping resolution	Coarse	Coarse	Coarse	Coarse	Fine	Fine	Fine	Fine
Replicated measurements	Not possible	Possible	Not possible	Possible	Possible	Possible	Possible	Possible
Detection of smaller effect QTLs	Low	Low	Low	Low	Low	High	High	High

**Table 3:** Basic differences among various marker based selection schemes.

	MABC	AB-QTL	MARS	GS
Test population	Mapping population (family-based)	Mapping population (family-based)	Mapping population (family-based)	Training Population (family-based/diverse germplasm)
Relies on	Linkage	Linkage	Linkage	Genome wide LD
QTL discovery and introgression	Two different populations	Same population	Same/two different populations	Two different populations
Historical phenotypic data	Not required	Not required	Not required	Required
Efficient in capturing	Few major QTLs	Exotic QTLs	Several minor QTLs	Genome wide QTLs
Statistical analysis	Simple	Simple	Relatively simple	Computationally challenging
Marker-trait establishment	Not performed	Performed	Not necessary	Not performed
A prior QTL information	Required	Not required	Not required (ad hoc index can be used)	Not required
Marker genotyping	Target markers only	Several markers	Several markers	Genome wide
Markers used for	Selection	Selection	Selection and intermating	GEBV calculations
Genetic gains (compared to PS)	Low	Moderate	Moderate to high	Highest

**Table 4:** List of some recently developed phenotyping platforms/software tools.

Name of platform/software tool	Link	References
CLID	http://ecotheory.biology.gatech.edu/	—
DIRT	http://ecotheory.biology.gatech.edu/	—
GERMINATOR	—	[119]
GiA Roots	http://giaroots.biology.gatech.edu/	[120, 123]
GlyPh	—	[124]
GROWSCREEN	http://www.fz-juelich.de/ibg/ibg-2/EN/methods_jppc/GROWSCREEN/_node.html	[120, 125]
HTPheno	http://htpheno.ipk-gatersleben.de/	[120, 126]
PHENODYN	http://bioweb.supagro.inra.fr/phenodyn/	[127]
PhenoPhyt	https://vphenodbs.rnet.missouri.edu/PhenoPhyte/index.php	[128]
PHENOPSIS	http://bioweb.supagro.inra.fr/phenopsis/	[120, 129]
Phenoscope	http://www-ijpb.versailles.inra.fr/fr/plateformes/ppa/index.html	[130]
Phytomorph	http://phytomorph.wisc.edu/	[119, 131]
RootLM	http://www.plant-image-analysis.org/software/rootlm	[119, 132]
RootNav	http://sourceforge.net/projects/rootnav/	[133]
RootTrak	http://sourceforge.net/projects/rootrak/	[134]
RosetteTracker	http://telin.ugent.be/~jdvylder/RosetteTracker/	[135]
Shovelomics	http://plantscience.psu.edu/research/labs/roots/methods/field/shovelomics/shovelomics	[136]
SPICY	http://www.bioss.ac.uk/people/yu/spicy/	[137]
TraitMill	http://www.cropdesign.com/tech_traitmill.php	[120, 138]
Trayscan	http://www.medealab.de/englisch/e_biovision_applications_classify_trays.html	—
WIWAM	http://wiwam.be/	[139]
